# Modeling the Impact of Social Determinants of Health on HIV

**DOI:** 10.1007/s10461-021-03399-2

**Published:** 2021-09-03

**Authors:** Joseph W. Hogan, Noya Galai, Wendy W. Davis

**Affiliations:** 1 Department of Biostatistics, Brown University School of Public Health, Providence, RI USA; 2 Department of Epidemiology, Johns Hopkins Bloomberg School of Public Health, Baltimore, MD USA; 3 Department of Statistics, University of Haifa, Mt Carmel, Israel; 4 Department of Prevention and Community Health, the George Washington University Milken Institute of Public Health, Washington, DC USA

**Keywords:** HIV, Social determinants of health, Mechanistic models, Causal models, Statistical models

## Abstract

There is growing evidence for the key role of social determinants of health (SDOH) in understanding morbidity and mortality outcomes globally. Factors such as stigma, racism, poverty or access to health and social services represent complex constructs that affect population health via intricate relationships to individual characteristics, behaviors and disease prevention and treatment outcomes. Modeling the role of SDOH is both critically important and inherently complex. Here we describe different modeling approaches and their use in assessing the impact of SDOH on HIV/AIDS. The discussion is thematically divided into mechanistic models and statistical models, while recognizing the overlap between them. To illustrate mechanistic approaches, we use examples of compartmental models and agent-based models; to illustrate statistical approaches, we use regression and statistical causal models. We describe model structure, data sources required, and the scope of possible inferences, highlighting similarities and differences in formulation, implementation, and interpretation of different modeling approaches. We also indicate further needed research on representing and quantifying the effect of SDOH in the context of models for HIV and other health outcomes in recognition of the critical role of SDOH in achieving the goal of ending the HIV epidemic and improving overall population health.

## Introduction

The social determinants of health (SDOH) have been defined by the World Health Organization (WHO) as “the conditions in which people are born, grow, work, live, and age, and the wider set of forces and systems shaping the conditions of daily life” such as “economic policies and systems, development agendas, social norms, social policies and political systems”[1]. The WHO notes that these circumstances “are shaped by the distribution of money, power and resources” and are largely responsible for health inequities [1]. In the last two decades or so, investigators have come to appreciate that an individual’s vulnerability to disease is multi-factoral and subject to community, societal and environmental determinants [2].

Global pandemics such as the ongoing HIV pandemic, where SDOH play a central role in incidence and disease burden outcomes [3], present an important opportunity for utilizing models to inform investigators, practioners and policy-makers about how SDOH shape and impact epidemic and disease dynamics. The role of SDOH in the trajectory of the HIV epidemic has received increasing recognition, especially since the 2000s with a growing number of public health and epidemiologic studies designed to assess the role of SDOH through observational, intervention and randomized trial designs [4–6]. As the availability of data and newly developed measures around SDOH and inequitable health outcomes grows [7], so does the potential utility of disease and intervention models to illuminate the relationship between SDOH and indicators of individual and population health. Well-formulated models can be used to quantify and provide insight about the impact of planned or already implemented interventions at both the individual and community level.

This paper joins this AIDS and Behavior supplement, devoted to research conducted through the NIMH and NIAID funded RFA, Methodologies to Enhance Understanding of HIV-Associated Social Determinants [8], to offer a reflection on the specific ways in which models can be employed to elucidate relationships between SDOH and HIV outcomes. The paper provides a broad overview of two basic and foundational approaches, namely mechanistic and statistical models, and considers how two papers in this volume, that provide findings from modeling analyses, reflect the basic structures of these exemplars. While our review is divided thematically into mechanistic and statistical approaches to modeling, we recognize this distinction to be somewhat artificial as the approaches are not mutually exclusive. In fact the overlap can be substantial, and the coherent integration of methods that are traditionally labeled ‘mechanistic’ and ‘statistical’ is an active area of ongoing methodologic research. This paper speaks to the commonalities between mechanistic and statistical models and shows how they can be used to draw various kinds of inferences about the role of social determinants of health in HIV research.

Our discussion of these models is necessarily concise; hence, for comparison purposes, we focus on model structure, the sources of information that inform parameters (model inputs), and how the models are used to generate inferences (model outputs). Examples are given to illustrate the different modeling approaches. We discuss considerations for model choices and issues related to the generalizability of results from the various modeling approaches. Finally we describe connections between statistical and mechanistic models and give potential directions for future research.

## Model Structure and Some Reasons for Using Models

To fix ideas for comparing the models, it is hepful to introduce some basic notation that will be used throughout. We assume that all models in our discussion have the basic form Y = M(X, θ, ε), where Y is an outcome of interest, X are inputs or covariates, θ is a set of model parameters, ε represents random variation (e.g. random error), and M is a model that describes how the inputs and parameters are related to Y. This is a generic description but it will be useful in organizing our discussion. A familiar example of a model is the normal-error linear regression Y = α + βX + ε, where ε is a normally-distributed error term having mean zero and standard deviation σ. In this case, Y is the output, X is the input, θ = (α, β, σ) is the set of parameters, and ε captures the random variation in Y after accounting for systematic variation explained by X. The model is the normal probability distribution function where the mean is α + βX and the standard deviation is σ.^1^

Models have various types of uses and the ultimate choice of a model is based both on the information available and the intended use. Our focus here is on the use of models to understand the impact or role of SDOH on outcomes of interest such as the risk of HIV infection, and to understand the impact of interventions or policy changes that might be motivated by or targeted to considerations related to SDOH. It is also important to note that human interactions are central for understanding the dynamics of infectious diseases transmission and control. For example, suppose we are interested in the effect of needle exchange on HIV incidence in a population of injection drug users at risk for HIV. Consider a model of the form Y = M(X, A, θ,ε). In this case, Y is HIV incidence, A is needle exchange, X are individual- and community-level predictors of HIV incidence, and θ is a set of parameters that governs the relationship between A, X and Y via the model M.

There are two ways to use models to study this effect. The first is to use knowledge about the form of the model itself; that is, to use, for example, a set of mathematical equations that describes how HIV transmission takes place in a population having characteristics and behaviors captured by inputs X, and then to use that model to generate predictions of Y under different levels of A. In this case, the ‘inputs’ are knowledge about the mathematical form of the model (that is, model structure), and values of X that describe a population, or individuals in the population, including, for example, frequency of drug injection and likelihood of sharing injection equipment. An assessment of the effectiveness of needle exchange is made by simulating values of Y under different values of A; for example, simulating HIV incidence under assumptions that needle exchange is fully available (A = 1) or not at all available (A = 0). Such a mathematical model might also encode assumptions about how individuals interact with each other—information that would be difficult to obtain from observed data. Because this approach relies on the user specifying the mathematical form of the model that reflects the mechanism leading to HIV incidence—we call these models *mechanistic.* Mechanistic models also typically include assumptions about the values and distribution of some of the model parameters, often derived from knowledge or information gained from previous studies.

A second approach is to use what we call here *statistical* models, where individual-level data is used to estimate parameters of the model itself. In a statistical model, the model structure does not attempt to explicitly describe the underlying disease dynamics but rather the association between covariates and outcome, often assuming a general linear structure. In this case, we may have individual-level data on Y, A and X. For example, Rich et al. [9] recorded data on HIV incidence (Y) for individuals at outpatient treatment centers in two neighboring states (RI and MA) where, by law, access to needle exchange was substantially different. In this case, observed individual-level data are used to estimate the parameters (θ) of an instrumental variables model, which takes a much simpler form than the mechanistic model.

An instrumental variables model can be formulated as a simultaneous equations model with correlated error terms (one equation for the outome and a second for the exposure). In this context, the model would be fit under an assumption that any between-state differences in the outcome Y are assumed to be attributable solely to differences in needle exchange access. Rich et al. use this model to estimate the impact of access to needle exchange (A = 1 versus A = 0), on syringe re-use and sharing and demonstrate that access to needle exchange substantially reduces both. In fact a version of this model can be used to generate simulated outcomes under A = 1 and A = 0 as a way to assess the impact of needle exchange, which mimics how a mechanistic model might be used for the same purpose, a point we return to later in the paper. A key difference between the mechanistic model and the statistical model is that a single source of data is used to estimate the parameters of the statistical model, whereas multiple data sources might be used to determine fixed values or distributions of parameters for the more complicated mechanistic model. Additionaly, while mechanistic models attempt to represent multiple complexities or stages of a process leading to a health outcome Y, statistical models typically rely on simplified versions of data generating mechanisms.

In the next two sections we provide some additional description and concrete examples of mechanistic and statistical models and illustrate how they are used to assess the role of SDOH and to generate causal comparisons of interventions that are motivated by or closely related to SDOH. Where possible we make reference to the model structure described above.

## Mechanistic Models

Mechanistic models are rooted in a mathematical representation of the mechanism driving the process of interest. Mechanistic models used to characterize population dynamics of HIV infection are often formulated to capture the population structure, infection transmission dynamics and stages of disease progression. Our discussion of mechanistic models uses examples of compartmental and agent-based models that have been applied to model HIV in a variety of populations and that incorporate SODH.

A major motivation for using mechanistic models to study SDOH is the need to characterize a complex system or process that cannot be studied using a single source of data. Hence the model inputs and information about model structure, in terms of key parameters, values or functional form of key mathematical components, can derive from multiple sources [10]. For some parameters there may be little or no a-priori information available; these values are typically tuned or fixed using a calibration process. To calibrate the model parameters, simulations from a version of the model having fixed parameter values are compared to data on a the outcome of interest, such as HIV incidence over a period of time, measured in the target population. Optimization or grid-search methods can be used to identify the parameter values for which simulated outcomes from the model align with observed data. Importantly, for a given model, there may be more than one set of parameter values for which simulations from the overall model are consistent with the data used for calibration; in other words, the post-calibration parameter values may not represent a unique solution [11].

### Compartmental Mechanistic Models

Compartmental models of infectious disease dynamics provide a prototypical example of a mechanistic model. These mathematical models represent the process of interest assuming a structure that is driven by the definition of a set of disease-related states (compartments) and the rules that govern the transition between compartments. These models are often specified using a set of differential equations to describe for example transitions between different compartments that respresent disease states (e.g. susceptible, infected, recovered; or transitions between HIV disease states that reflect disease progression and are defined in terms of CD4 count and viral load [12].

Compartmental models can be specified to quantify the effects of demographic factors and SDOH at either the population or individual level including, for example, economic factors such as income distribution, educational attainment, and knowledge of disease risk, or to characterize a more refined representation of the disease process including incubation period, disease progression, treatment status and death [13]. Thus SDOH may be incorporated by defining compartments corresponding to specific sub-populations, by say education levels or race, or by allowing transition parameters to be influenced by social determinants such as access to care or stigma and discrimination. Dynamic compartmental models can be used to represent the changes in disease prevalence over time due to changes in transmission rates and or population-level dynamics such as migration patterns or proportion of antiretroviral treatment coverage.

A compartmental model that has been used for a variety of purposes is the Estimation and Projection Package Age-Sex Model (EPP-ASM) [14], implemented in the Spectrum software package [15]. This model is used in the paper by Jahagirdar et al. [16] in this supplement to derive country-specific estimates of HIV incidence over time in over 40 countries in Africa. Within the Spectrum model, incidence rate, which in our notation is the Y variable, is modeled as a function of several inputs that comprise the country-specific X variables: transmission rate among untreated HIV-infected individuals, HIV prevalence, proportion of HIV-positive individuals on ART, and the effectiveness of ART at reducing onward transmission. The model M(X, θ, ε) is a set of mathematical equations that describes how the X variables are related to HIV incidence; the values of the θ parameters have been derived from different sources. For a given set of inputs X, which the user needs to supply, the model can then be used to generate estimates or even simulations of the number of new cases that would be anticipated based on the model inputs. Notably the model inputs for this example are specified at the population level as opposed to the individual level.

Other examples of compartmental models that have been proposed to study HIV incidence and prevalence and their relationship to SDOH include Shannon et al. [17] who showed the impact of gender-based violence and discrimination of sex work on new HIV infections among female sex workers. Compartmental models can be also be used for post-hoc quantification of the contribution of individual components of a complex intervention by simulating hypothetical scenarios that are not likely to be reproducible in practice and would otherwise be difficult to isolate empirically. Examples include Nosyk et al. who demonstrated the impact of harm reduction services and ART coverage on averting new HIV infection in the population due to needle sharing [18].

### Agent-Based Mechanistic Models

Individual or agent based models (ABM), also termed micro-simulation models, can be viewed as higher-resolution versions of mechanistic models and can be used to characterize and simulate outcomes based on individual-level behavior. ABM treat each individual in the population as unique and can incorporate or represent information about relationships between individuals.

In compartmental models such as the Jahagirdar et al. HIV incidence model described above [16], heterogeneity in a population is introduced by division of compartments into smaller subgroups, such as by age or risk profile (e.g. men who have sex with men (MSM), drug users, or those with pre-existing conditions) where each subgroup has possibly different transition rates between states; that is, the compartmental model is developed at the population level and assumes that individuals within each compartment are homogeneous (a top to bottom approach). By contrast, individual-based or microsimulation models introduce heterogeneity at the individual level whereby each individual can be assigned unique characteristics and risk profiles and a personal pattern of contacts with other individuals in the population (a bottom-up approach). In an individual-level model for HIV incidence, for example, each individual has their own probability of transmitting and contracting the infection. In our generic model representation, Y denotes a new HIV infection for an individual (e.g. 1 if yes, 0 if no). The inputs X can include both individual-level and population- or stratum-level variables.

Like population-level mechanistic models, ABM typically require simulation solutions to produce a projected output and often utilize multiple sources of data to obtain information on the values of model parameters and their distribution. Within the ABM framework, SDOH such as education, socio-economic status, racial or gender minority affiliation can be incorporated in individual characteristics that compose a simulated population and in turn, determine the interactions with other individuals in the population. Simulated output from agent-based models, then, produce overall epidemic dynamics and selected health outcomes under different assumptions on population structure and inter-personal interactions.

Similar to compartmental models, ABM have beeen used to assess the potential effects of complex interventions that cannot be easily studied using a single cohort or source of data. For example, Marshall et al. [19] used an ABM to model HIV incidence among interacting individuals that included injection drug users, non-injection drug users and non-drug users in New York City between 1992 and 2002. The model formula can be written generically as Y = M(X, A, θ, ε), where X represent individual-level characteristics, A denotes a system-level intervention such as availability of needle exchange, and the ε term represents the stochastic or probabilistic component of the model. Marshall et al. used this model to simulate individual HIV infection status within each subpopulation under various system-level interventions and used the output to generate population- and stratum-level HIV incidence trajectories. By generating simulated outcomes under different configurations for the interventions—which in the model correspond to different versions of the A variable—they demonstrated that the combination of syringe exchange and provision of ART could produce substantial reductions in HIV prevalence among injection drug users. This finding mirrored the results from several empirical studies [20]. An important feature of this ABM is that the model itself was parameterized to allow interactions between individuals under different network assumptions. Although typically there are limited data available to verify whether population interaction assumptions are correct, the model can be used to generate simulations under various assumptions about network structure in order to quantify the robustness of findings about intervention effects.

An additional example of agent-based modeling is carried out by Brookmeyer et al. [21], who used simulations from an agent-based model to evaluate the impact of 163 different HIV prevention packages comprising varying combinations and intensities of four prevention measures: percent of eligible persons who receive ART, percent reduction in unprotected anal intercourse (UAI), percent of eligible persons accepting PrEP, and a variable capturing increase in HIV testing. In empirical studies, these had only been examined one at a time. The analysis followed two steps: in the first step, simulation of data from an agent-based model where the population starts out with initial values of HIV prevalence, knowledge of HIV status, testing frequency, and frequency of sexual risk behavior. This model also makes assumptions about frequency of sexual contact based on an assumed social network structure, and about the probability of HIV transmission per contact. Individual-level outcomes are simulated forward in time.

In the second step, a regression model is applied to the simulated data to estimate the impact of varying the percent intensity of each prevention measure. The regression model used by Brookmeyer et al. [21] includes covariates representing percent uptake of the prevention measures listed above; fixing these values in different combinations encodes the 163 distinct prevention packages. Aside from the assumptions used to generate the synthetic data from the ABM, another critical set of assumptions is the specification of the impact of each intervention in the regression model. Brookmeyer et al. assume that, on the logit scale, the effects of ART uptake and UAI are additive and that the impact of PrEP is dependent on the rate of HIV testing. SDOH in this model were incorporated as factors impacting various prevention strategies such as ART and PrEP coverage and condom use, that are influenced by societal and cultural norms, stigma and discrimination as well as empowerment.

An advantage of this approach is that we can potentially gain insights about combinations of interventions that typically can only be tested one at a time in a randomized trial (if they can be tested at all). A key limitation is the strong dependence on modeling assumptions: the data generated by the ABM are synthetic, and the estimated impact of combination interventions depends on how each intervention is parameterized in the second-step regression model.

## Statistical Models

Statistical models tend to have a less complex mathematical structure, and typically are used to draw inferences based on a single source of individual-level data from a target population (e.g. a cohort study or clinical trial). The most commonly used statistical models are regression models, which quantify associations between the inputs X and the outcome variable Y with the regression coefficients β. While it is possible to conduct a statistical analysis of a complex mathematical model, statistical models per se are not typically designed to represent population-level mechanistic processes. In contrast with mechanistic models that rely on calibration to tune parameter values, in statistical models the parameter estimates are usually derived by fitting the model to a single source of data using techniques such as maximum likelihood. For a defined statistical model and a given dataset, the parameter estimates resulting from the fitting process are typically unique. Under certain circumstances, statistical models also can be used to generate causal inferences and to assess the impact of interventions. One approach to accomplishing this goal is the g-computation algorithm, which serves as a conceptual connector between statistical and mechanistic models. We describe the g-computation algorithm below using the analysis conducted by Stoner et al. for this supplement, as an example [22].

### Regression Models

The goal of a statistical regression model is to characterize explained and unexplained variation in one or more outcome variables Y based on data drawn from a target population. The explained variation is assumed to depend, through a regression function, on a set of predictor or explanatory variables X; for example, a regression model can be used to characterize variation in a disease outcome as a function of multiple explanatory risk factors such as age, gender, risk behaviors and factors related to SODH. Regression models provide estimates of the association between the explanatory variables and disease outcomes in the form of differences in means or risk, risk ratios, odds ratios, and hazard ratios, and can also be used to assess the potential impact of determinants via measues such as population attributable fraction. Regression models can be used to assess the effect of an SDOH or policy at the individual level as well as the community level.

An example of this kind of analysis is given by Kemp et al., who use a multilevel regression model to demonstrate the impact of ongoing experiences of HIV stigmatization on increased viral load among African-American women in primary HIV care [23]. Multilevel models are generalizations of standard regression models in that they build in error terms at each level of a clustering hierarchy; they are sometimes called random effects models, mixed effects models, or mixed models. They also can be used to estimate separately the effect of a covariate at each level of the hierarchy. By using a multilevel model that decomposes the overall effect of stigma on viral load, Kemp et al. were able to show that within-person changes in stigma over time do not lead to subsequent changes in viral load (within-individual effect), but that individuals having higher levels of stigma on average also have higher viral load on average (between-individual effect) [23]. This distinction between within- and between-individual effects is critical not only to understanding the mechanism by which SDOH might operate, but also for understanding how interventions might be designed.

The analysis conducted by Jahagirdar et al. in this volume borrows techniques from both regression modeling and mechanistic modeling [16]. In their model, simulated rates of HIV infection in over 40 countries in Sub-Saharan Africa, derived from the EPP-ASM model described above, are used as the outcome in a multilevel regression model to assess the impact of individual- and community-level SDOH on HIV risk [16]. The incidence rates predicted from EPP-ASM showed an overall decline in HIV incidence between 2000 and 2015. They then use a multilevel regression model to characterize variation in the predicted HIV incidence over time accounting for both between- and within-country variation in incidence, and the dependence of incidence on country-level SDOH covariates. Jahagirdar et al. use this approach to identify SDOH and other variables that explain the highest percentage of variation in country-specific HIV incidence rates. Average number of education years per capita and country-specific spending on HIV emerge as the factors explaining the greatest amount of variation in HIV incidence rates between countries [16].

### Causal Models Derived From Regression Models

While regression models are formulated to capture the effect of predictor variables on an outcome of interest, the effect cannot generally be interpreted as causal. In this section, we discuss the construction and interpretation of causal structural models, and illustrate the use of one such model by Stoner et al. in this supplement to quantify the effect of child support grants (CSG) on HIV incidence among adolescent girls and young women [22].

Causal structural models are specified in terms of random variables called potential outcomes. For a two-level exposure or intervention, such as receipt of cash transfer or not, a causal model assumes that each individual has two potential outcomes: the outome that would be realized if the intervention is received, and the other that would be realized if not received. Unlike with other sorts of models, the potential outcomes formulation of the causal model assumes that both variables exist for each individual, even though only one can be observed [24]. (Potential outcomes are sometimes referred to as counterfactuals because for each individual, we can only observe the potential outcome corresponding to the actual exposure received; the other one is counterfactual.) From a statistical perspective, the goal is to draw inference about the difference or ratio of means between these two potential outcomes using observed data. The fundamental challenge is that, for each individual, only one of the potential outcomes can be observed. The process for drawing inference about causal effects from observed data can be driven either by design—i.e. by randomizing individuals to exposure or no exposure in a clinical trial design—or by analytic methods that are designed to balance confounding variables that make selection into the exposed and unexposed groups systematically different.

A design-based approach to causal inference is to randomize individuals to the exposure. Under randomization, for each individual, we are equally likely to observe either outcome and we can use the observed outcomes under each condition to estimate differences or ratios of the outcome of interest at the population level. If data were collected in an observational study instead, it is important to remember that girls and women who are at higher risk for HIV infection may also be more likely to receive cash transfers. Statistical methods used to estimate causal effects in settings where the exposure is not randomized are therefore designed to mimic randomization in some way by accounting for possible confounder imbalance. This can be accomplished by reweighting the sample according to inverse probability of receiving treatment [25, 26], matching those who receive treatment to control individuals with similar observed characteristics [27, 28], or making model-based adjustments.The propensity score, a summary of the probability of being exposed as a function of covariates, plays a fundamental role in many of these approaches [29]. There is a vast literature describing these methods and many others; a full review is beyond the scope of this discussion. Instead we focus on the g-formula method [30] used here by Stoner and colleagues.

Stoner et al. investigate the causal effect of cash transfer on HIV infection among adolescent girls and young women (AGYW) [22]. In the simplest formulation of their causal model, there are two potential outomes for each person: HIV infection status under the scenario that CSG is received, and HIV infection status under the alternate scenario that CSG is not received. Stoner et al. use a more complex version where CSG can vary over time, and have different intensities [22].

Implementation of the g-formula has many similarities to agent-based modeling [31]. The g-formula can be used to simulate potential outcomes under different versions of an intervention that are fixed by the investigator; the outcomes simulated under different scenarios are then used to quantify intervention effects. The process of simulation in the parametric g-formula bears similarity to how simulation-based inferences are generated in mechanistic models in the sense that individual component models are used to generate simulated outcomes of interest, such as HIV incidence, under different versions or intensities of an intervention. A key difference is that for the g-formula, the component models used to generate simulated potential outcomes are statistical models^2^—oftentimes regression models—that have been fit to a single source of observed data drawn from a population of interest. In practice, therefore, simulations from the g-formula typically derive from component models that are less mathematically complex but have closer fit to a representative sample of observed data. Mechanistic models are more complex representations of disease dynamics, but the data come from sources that may be less representative of a specific population.

Stoner et al. use the g-formula to examine receipt of CSG and other interventions [22]. At each time point, there are data available on HIV incidence (Y), receipt of CSG (A), and confounding variables (X). In this example, implementation of the g-formula proceeds in three steps: first, for each time point, fit a regression of HIV incidence as a function of CSG (yes/no) and the confounders. This regression, which can be represented in the format Y = M(X,A,θ, ε) where X are the confounders and A is the intervention, is the component model on which simulations of potential outcomes under different intervention combinations will be generated. Second, once the models are fitted to observed data, fix covariate values X to represent the population of interest, and fix the intervention sequence (the value of A at each time point) for which HIV incidence is to be estimated. Third, for this fixed version of the intervention sequence, generate simulated or predicted values of HIV incidence from the fitted regression models. In this way, the fitted regression models are playing the same role as the mechanistic models when it comes to simulating outcomes under different intervention scenarios.

Using this basic approach, Stoner et al. [22] can compare various intensities of CSG, such as all-versus-none receipt of CSG and all-versus-observed receipt of CSG. The latter comparison quantifies the effect of increasing the observed CSG coverage (around 75%) to having everyone receive CSG. With suitably rich data, this general strategy can be used to quantify the impact of more complex interventions or, as Stoner et al. do, to compare the interactive effect of interventions with other factors [22]. Their analysis shows the potential for combining monthly child support grants with interventions to increase parental care and reduce depression can lead to substantial reductions in HIV incidence among AGYW, and that these effects are not realized through cash grants alone [22].

## Discussion

As both the decades-old HIV global pandemic and the more recent SARS-CoV-2 pandemic demonstrate, SDOH can play a central role in the transmission, morbidity and mortality of an infectious disease. The models described here offer a range of tools that can help to elucidate the interplay between social and structural determinants and the expression of an infectious disease among individuals as well as the public health burden in the population. The presentation here is a broad overview of different established approaches to modeling disease outcomes, with examples that focus on HIV and SDOH. This is not meant to be a comprehensive toolbox. Models can offer insights into how an intervention might function at an individual level such as the causal model shared by Stoner et al. [22] as well as the societal level, such as the hybrid model developed by Jahagidar and colleagues [16], using information from mechanistic models as inputs to a statistical regression analysis of country-level exposures. These models allow for a simulation of outcomes under different versions of, intensity of, or combination of interventions that would be difficult to gain through designed studies such as randomized trials. While the ultimate goal of all the models discussed in this paper is to better understand the causal relationship between exposures and outcome, models that implement simulations of outcomes under various exposures address causality more directly compared to simpler regression models. In the case of Stoner et al., the availability of high quality individual-level data from an intervention trial allowed the investigators to examine complex versions of a time-varying intervention and its potential interaction with other factors [22]. For Jahagidar et al., the generation of country-level comparisons offered insights into the impact of programs and policies far beyond the potential scope or feasibility of any designed interventional study [16].

Many factors contribute to the validity of model-based inferences about SDOH. In this paper we have focused on model specification, model inputs (i.e. the data or information used to generate outputs from the model), and the use of models to assess the impact of interventions. Both mechanistic and statistical models rely on a representation of the underlying data generating process given in mathematical and probabilistic terms. Many compartmental models, for example, are written in terms of differential equations that describe the probability of transition from one compartment or disease state to the next; regression models describe variation of an outcome in terms of explained and unexplained variation, where explained variation is the regression function and unexplained variation, quantified by the error term, follows a probability model such as the normal distribution.

In nearly all cases, even the most complex model will not be an accurate or complete representation of the system or phenomenon that it is being studied. The models described in this paper, for example, are designed to characterize outcomes such as HIV infection as a function of SDOH and possible interventions, but do not address the dynamic processes that give rise to the SDOH themselves. However, models offer a useful insight into the potential impacts of selected factors, including various SDOH, on disease outcomes. Models must strike a difficult balance between interpretability, face validity, and fidelity to an observed-data process. The first two of these criteria are largely subjective. Regarding fidelity to observed-data processes, mechanistic models and statistical causal models are themselves built up from smaller submodels. While these submodels can be checked for lack of fit against observed data, the larger model relies on assumptions that tie the submodels together and typically cannot be validated against a single sample of data.

The validity of model-based inferences also depend on inputs. Parameter values for mechanistic models typically are informed by multiple sources that may even be derived from different populations. Statistical models tend to rely on single samples of data drawn from the target population. This distinction can be important. As Murray et al. demonstrate in a comparison of agent-based and statistical models for estimating causal effects, even when both models are perfect representations of the underlying system, using inputs from different populations—as is done with ABM and other mechanistic models—can induce unintended confounding and biased estimates of causal effects [31]. This is an especially important consideration when estimating effects of SDOH, particularly if the SDOH inputs are derived from substantively different populations than the confounding variables.

Uncertainty associated with model-based inferences has many sources. In statistical models, the most obvious is sampling variation, captured in terms of standard errors and confidence intervals. For mechanistic models, parameter inputs derived from published studies carry uncertainty because they usually correspond to estimates from other studies, which themselves have associated standard errors. This uncertainty can be represented by using distributions instead of fixed parameter values as model inputs, as is done for the Thembisa model of the HIV epidemic in South Africa [32]. The Bayesian approach to inference treats the model parameters as random variables. It requires specification of a probability model for the outcomes (what we have been referring generically to as M(X, θ, ε)) and prior distributions for the parameters θ in the probability model; inference is based on the posterior distribution of the parameters given the observed data. Posterior variation in the parameter values and model predictions reflects both the prior uncertainty about parameter values and sampling variability in the observed data. Bayesian methods can be used to fit both mechanistic and statistical models; however, they are particularly useful for mechanistic models that can be specified in terms of a likelihood function because they provide a formal way to encode existing information about model parameters via the prior distribution. An outstanding and timely example is the Bayesian model developed by Flaxman et al. [33] to quantify the impact of non-pharmaceutical interventions on COVID-19 in Europe.

Untestable assumptions are an often overlooked source of uncertainty. Statistical causal models rely by necessity on the assumption that all relevant confounders have been measured (the ‘no unmeasured confounding’ or ‘treatment ignorability’ assumption), but there is no way to verify whether or not this assumption holds. Uncertainty about untestable assumptions should be examined in sensitivity analyses, which serve to quantify the robustness of inferences from causal models—whether statistical or mechanistic. Sensitivity analyses can take many forms [34, 35], and a critique of assumptions about both mechanisms and confounding can be guided by the use of a graphical model [36].

Finally an often overlooked source of uncertainty is the quality of measurement and design. The value of any given model is tied to the quality of its input and the rigor of its design [37]. The interpretation of modelled epidemiological scenarios must include a critical assessment of the accuracy of measurement and ascertainment of exposure and outcomes, and how any data source is calibrated and considered against other inputs.

On a more general note, as Geffen and Welte explain, the nature of a model world, namely a “conceptual realm”, which is constructed around an understanding of “real world processes," should be comprehensible [38]. They argue that while the technical construction of a model may be complex and understood by relatively few, model worlds should be accessible to all that work within that domain [38]. For compartmental or micro-simulation models, users should understand the basic rationale behind the mathematical model used to represent population dynamics and the validity of parameter values that populate the model. A full systematic reporting of the evidence synthesis and model assumptions is critical for models to be useful tools for policy decision making [14]. For causal models, users should understand the nature of key assumptions such as ‘no unmeasured confounding’ and how they are applied in the specific context.

One way to ensure that models are comprehensible and that they reflect a theoretically valid model world is to further integrate the technical development of models with longstanding and rigorous research on the SDOH. The rich theoretical foundation of research on the SDOH and their association with disease processes and outcomes should also be reflected in any mathematical or statistical model development. This integration of theoretical research in sociology, especially of more complex concepts such as systemic racism and discrimination, with statistical model development presents a real challenge. Developing valid measurement of SDOH at both the individual level, assessing exposure to and impact of social determinants, as well as quantifying differences at the community level, such as police or justice system discrimination, are critical. Models of SDOH will also be strengthened by the use of high-quality inputs. Collaborations with investigators working in social behavioral science can ensure that modellers use well regarded measures and inputs as ingredients in the models they construct. There is a broad need then for models that are grounded in social behavioral theory and draw on data from a diversity of sources with indicators that are appropriate for the model’s proposed context and use. As epidemics unfold and mature, there is a significant opportunity for the application of models as we work to understand how communities and policies impact disease outcomes and how shifting realities in individual circumstance may support or hinder optimal outcomes.

## Data Availability

Not applicable.

## Footnotes

1 The model itself is written mathematically as p(Y | X,α,β,σ) = { (2)}^−1^ exp{—(Y—α—βX)^2^ / (2σ^2^)}, which describes the relative probability of observing the value Y for a fixed value of X.

2 Strictly speaking, the g formula does not require the component models to fitted statistical models. However in epidemiologic practice this is the most common method of applying the g formula.
